# Collaborative Filler Network for Enhancing the Performance of BaTiO_3_/PDMS Flexible Piezoelectric Polymer Composite Nanogenerators

**DOI:** 10.3390/s22114181

**Published:** 2022-05-31

**Authors:** Ayda Bouhamed, Nathanael Jöhrmann, Slim Naifar, Benny Böhm, Olav Hellwig, Bernhard Wunderle, Olfa Kanoun

**Affiliations:** 1 Measurement and Sensor Technology, Faculty of Electrical Engineering and Information Technology, Technische Universität Chemnitz, Reichenhainer Straße 70, 09126 Chemnitz, Germany; slim.naifar@etit.tu-chemnitz.de (S.N.); olfa.kanoun@etit.tu-chemnitz.de (O.K.); 2 Materials and Reliability of Microsystems, Faculty of Electrical Engineering and Information Technology, Technische Universität Chemnitz, Reichenhainer Straße 70, 09126 Chemnitz, Germany; nathanael.joehrmann@s2002.tu-chemnitz.de (N.J.); bernhard.wunderle@etit.tu-chemnitz.de (B.W.); 3 Functional Magnetic Materials, Faculty of Natural Sciences, Technische Universität Chemnitz, Reichenhainer Straße 70, 09126 Chemnitz, Germany; benny.boehm@physik.tu-chemnitz.de (B.B.); olav.hellwig@physik.tu-chemnitz.de (O.H.)

**Keywords:** PDMS/BaTiO_3_ nanocomposite, MWCNTs, flexible piezoelectric nanogenerators, biomechanical energy harvesting, temperature dependency

## Abstract

Wearable sensors are gaining attention in human health monitoring applications, even if their usability is limited due to battery need. Flexible nanogenerators (NGs) converting biomechanical energy into electrical energy offer an interesting solution, as they can supply the sensors or extend the battery lifetime. Herein, flexible generators based on lead-free barium titanate (BaTiO_3_) and a polydimethylsiloxane (PDMS) polymer have been developed. A comparative study was performed to investigate the impact of multiwalled carbon nanotubes (MWCNTs) via structural, morphological, electrical, and electromechanical measurements. This study demonstrated that MWCNTs boosts the performance of the NG at the percolation threshold. This enhancement is attributed to the enhanced conductivity that promotes charge transfer and enhanced mechanical property and piezoceramics particles distribution. The nanogenerator delivers a maximum open-circuit voltage (V_OC_) up to 1.5 V and output power of 40 nW, which is two times higher than NG without MWCNTs. Additionally, the performance can be tuned by controlling the composite thickness and the applied frequency. Thicker NG shows a better performance, which enlarges their potential use for harvesting biomechanical energy efficiently up to 11.22 V under palm striking. The voltage output dependency on temperature was also investigated. The results show that the output voltage changes enormously with the temperature.

## 1. Introduction

Nowadays, energy harvesting technologies are facing an immense breakthrough owing to the increase of sophistication of our society using Internet of Things (IoT) technology. Therefore, millions of sensors are used that require continuous charging [1,2,3].

To this aim, the replacement of batteries with a continuous power source is becoming inevitable and indispensable in providing continuous environment and human health monitoring.

Additionally, the environmental concern has also pushed for efficient and eco-friendly generating energy sources. Therefore, the demand for energy harvesting technologies that can collect ambient energy such as wind energy, solar energy, and mechanical energy is growing [1,2,3]. Among all these sources, the ambient energy of mechanical vibrations is one of the most abundant sources that can be converted to usable electrical energy [4,5,6]. Various principles exist to harvest wasted mechanical energy as electricity, including the piezoelectric and triboelectric principles [7,8]. Both principles can efficiently harvest energy in low-frequency environments and can be lightweight. However, a triboelectric energy harvester has some limitations, such as lower durability caused by structural changes over time and post-stress conditions [9]. For this reason, piezoelectric energy harvesters are more preferred.

Among piezoelectric energy harvesters, polymer nanocomposite based piezoelectric energy harvesters attract great attention because of the possibility to convert vibrational and mechanical energy sources from human activities, such as pressure, bending, and stretching motions into electrical energy. Recently, different piezoelectric nanomaterials have been used to develop flexible nanogenerators (NGs) such as lead zirconate titanate (PZT), zinc oxide (ZnO), barium titanate (BaTiO_3_), or in poly (vinylidene fluoride) (PVDF) [10,11,12]. Between all these materials, PZTs have been frequently used, owing to their high piezoelectric properties and thermal stability [13]. However, the toxicity of lead led researchers to consider and use lead-free piezoceramic with comparable piezoelectric properties to PZT. Recently, BaTiO_3_ is explored for the realization of nanogenerators for different applications, including medical [14].

In fact, BaTiO_3_ is one of the promising lead-free piezoceramic due to its very high piezoelectric constant d_33_ > 200 pC/N, their perovskite crystal structure that leads to a high dielectric constant (100–11,000), and biocompatibility. BaTiO_3_ piezoelectric nanoparticles are preferred to be embedded into a soft polymer matrix to fabricate simple, scalable, and wearable piezoelectric nanogenerators [14].

For example, Shin et al. [15] reported about a highly performant flexible piezoelectric composite composed of BaTiO_3_ and poly-(vinylidene fluoride-co-hexafluoropropylene) (PVDF-HFP), which could achieve a maximum output voltage and current under cyclic bending in the range of 5 V to 750 nA, respectively. In another work, Yan and Jeong [16] developed flexible composites with different orientations of BaTiO_3_ nanofibers in a polydimethylsiloxane (PDMS) polymer. The nanogenerator with vertically aligned BaTiO_3_ nanofibers exhibited a high piezoelectric performance with an output power of 0.1841 μW under very low mechanical stress around 0.002 MPa.

Several works have illustrated that the performances of nanogenerators can be significantly improved by keeping them under a high electric voltage field of several kV for a very long period of time in order to orient the crystal domains of the piezoelectric material and to align the piezoelectric dipoles into an identical direction. Lin et al. [17] fabricated a stretchable piezoelectric nanogenerator using BaTiO_3_ /PDMS, which was polarized at an ambient temperature by applying an electric field of 80 kV/cm for 12 h. The nanogenerator delivered an open-circuit voltage and short-circuit current of 5.5 V and 350 nA, respectively. However, this process is long and unsafe. Intensive efforts have been made to improve the performance by introducing different carbon materials, such as carbon black, graphene, and carbon nanotubes (CNTs) [18,19,20,21].

Luo et al. [18] developed high-performance flexible piezoelectric nanogenerators containing a 30 wt.% BaTiO_3_/PDMS/3.2 wt.% carbon black composite film. The performance was enhanced by 143% compared to the nanocomposite without carbon black.

Additionally, many other studies have doped CNTs within the BaTiO_3_/PDMS composite. In general, the use of CNTs as filler leads to improving the output voltage, as they serve as nano-electrical bridges. Yan et al. [22] reported that the addition of 2 wt.% multiwalled carbon nanotubes (MWCNTs) to a nanogenerator with 40 wt.% BaTiO_3_ leads to an enhanced output performance of the nanogenerators due to the improvement of both the electrical and dielectric properties. Park et al. [12] demonstrated that the addition of single-walled carbon nanotubes (SW-CNTs) to PDMS/BaTiO_3_ can lead to a higher output voltage of ~3.2 V compared to the one containing both SW-CNTs and reduced graphene (rGO) oxide. This can be explained with the enhanced electrical properties of SW-CNT that can significantly influence the output performance. Furthermore, the NG with rGO shows less performance caused by the difference in the degree of mixing of both kinds of reinforcements, which leads to geometrical difference between the SW-CNT networks and the laminated rGO structures.

Importantly, the highest performance of nanogenerator filled with conductive particles were found to be at a critical loading of the filler known by the percolation threshold, where there was a sudden increase on the electrical conductivity in the nanocomposites. The percolation threshold depends greatly on the filler geometry and state of the filler dispersion in the polymer matrix. For a polymer composite containing spherical conductive particles, e.g., carbon black, the percolation thresholds were usually found to be very high, which enormously minimized the flexibility and increased the cost of the final composite. In order to tackle this problem, several approaches have been proposed by including conductive fillers with a higher dimension, such as one-dimensional (1D) fibers as MWCNTs or two-dimensional (2D) plates as graphene nanoplates were employed to ensure lower percolation thresholds [23].

A low percolation threshold can be achieved, usually with use of one-dimensional (1D) carbonaceous fibers such as MWCNTs. However, the main challenge is to avoid their agglomeration caused by their high surface area and Van der Wall attractive forces.

Therefore, efforts were devoted towards minimizing the percolation threshold by optimizing the processing conditions.

Several approaches have been followed by researchers to optimize the electrical conductivity and reduce the percolation threshold in polymer composites, including optimization of the processing conditions such as mixing temperature and speed or by involving powerful processes that are able to unbundle the agglomeration of nanoparticles, such as sonication or the calendaring process [24,25].

In this work, we explored the potential of polymer composites based on lead-free ceramics BaTiO_3_ to realize a wearable and flexible piezoelectric nanogenerator with less piezoceramics concentrations and enhanced performance without any additional poling processes. To achieve a cost-effective NG with improved performance, composite films containing different ratios of BaTiO_3_ piezoelectric nanoparticles in a polydimethylsiloxane (PDMS) polymer matrix were investigated through electromechanical measurements using a vibration shaker. Then, MWCNTs were dispersed into the BaTiO_3_/PDMS composite with a low concentration ranging from 0.3 wt.% to 1 wt.% to boost its performance. The distribution of nanoparticles was also examined via scanning electron microscope (SEM) and X-ray diffraction (XRD). The impact of the addition of MWCNTs was also addressed by evaluating and comparing the piezoelectric performance, as well the electrical and mechanical properties of the nanocomposites. The effects of layer thickness on the performance of the nanogenerator were also examined, and the potential of the realized NG for biomechanical energy harvesting was verified in ambient conditions and at different temperatures.

## 2. Materials and Methods

### 2.1. Materials

In this study, two different reinforcements were used: BaTiO_3_ and MWCNTs. The BaTiO_3_ particles have a high purity and 99.5% trace metals basis. They were purchased from Sigma Aldrich with a dimension less than 2 µm. As well, MWCNTs were purchased from Sigma Aldrich with an outer diameter of 6–9 nm and length of 1 µm. Concerning the polymer matrix, it consists of a soft polymer polydimethylsiloxane (PDMS—Sylgard 184) that was purchased from Dow Corning, GmbH.

### 2.2. Fabrication Process of the Piezoelectric Nanogenerator

**Figure 1:**
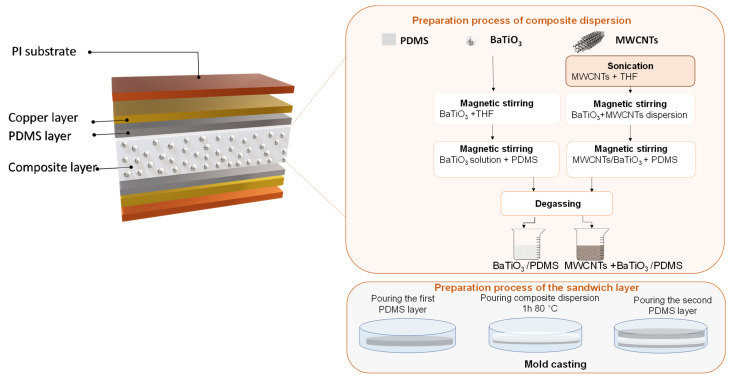
Flowchart of the fabrication process of different types of nanocomposites with an illustration about the structure of the final nanogenerator.

To realize a flexible piezoelectric nanogenerator based on the BaTiO_3_/PDMS composite, two layers of Kapton polyimide (PI) flexible substrate were used, in which the copper layer was coated in it to serve as an electrode. A laminate flexible composite was placed between the two sides. This laminate composite consisted of three layers composed of two dielectric layers of PDMS and a composite layer, which was deposited in between to form a sandwiched structure, as shown in Figure 1.

To produce the sandwiched structure, we start first by the fabrication of piezoelectric composites. Different composite compositions were made in this work, as summarized in Table 1. To prepare the composites, BaTiO_3_ was mixed in different ratios from 10 wt.% to 40 wt.% with tetrahydrofuran (THF) using a magnetic stirrer for 1 h, as shown in Figure 1. Then, the required amount of soft polymer polydimethylsiloxane (PDMS—Sylgard 184) was added and mixed for 2 h at 70 °C.

As the effect of the addition of MWCNTs on the nanogenerator performance is also addressed in this work. The preparation of the hybrid nanocomposite was approximately similar to the process used before, as shown in Figure 1. Due to their small size and large surface area, MWCNTs was firstly dispersed in THF using a horn sonicator Sonoplus HD 7300 for 15 min at 30% amplitude to unbundle the MWCNT agglomerations. To form the hybrid composite, the process presented in Figure 1 was followed.

After mixing, all dispersions were kept for the degassing process in the vacuum chamber before deposition. In meanwhile, the first PDMS layer was prepared and deposited in a glass mold to be cured for 10 min at 150 °C. Subsequently, nanocomposite material was deposited and cured at 80 °C for 1 h, followed by the deposition of the second PDMS layer.

In fact, these two dielectric layers are made to avoid charging in the electrodes, as well as to protect the composite layer during peeling from the mold to be damaged.

### 2.3. Nanocomposites and Nanogenerators Characterizations

The performances of the nanogenerators were investigated by means of an experimental setup consisting of a voltage generator, a shaker, and a digital oscilloscope for the acquisition of the output signal.

A fixed and harmonically distorted mechanical load of a 30 Hz frequency and having an RMS value of around 0.1 N were used to determine the optimal material composition.

To determine the generated output power, variable load resistances ranging from kilo Ohms to Mega Ohms were used to characterize the output power of the nanogenerator. To that end, the power output (*P*) of the piezoceramic composite was determined by measuring the voltage across a variable resistance placed in parallel to the nanogenerator, where the consumed power could be calculated as the result of the square of the output voltage divided by the value of the load resistance, as demonstrated in Equation (1):

(1) $$P=\frac{V_{out}^{2}}{R_{ext}}$$

To investigate deeply the uniformity of piezoceramics particles distribution and the influence of the addition of carbon nanotubes on the quality of the nanocomposite layers, scanning electron microscopy measurements were conducted on the cross-section using Zeiss Auriga 40. To prepare the samples for cross-sectional SEM observation, small samples of 0.5 × 0.5 cm were cut using a lever cutter tool. Then, all the samples were coated with a very thin Au layer in order to avoid the charging effect. The images were taken at 30 kV with a working distance of 2 mm using the InLens SE-Detector (secondary electrons).

In parallel, tensile tests were conducted for some samples using the universal tensile testing machine Instron ElectroPuls E10000 at a rate of 3 mm/min in order to determine the mechanical properties of the composites. To this aim, the specimens were prepared with a standard dog bone of ISO37 and had a very small size with an overall length of 35 mm, gauge length l_0_ of 10 mm, and width of 2 mm.

As well, the crystalline structure of the different prepared composites was examined using X-ray diffractometer Rigaku SmartLab with Cu-K_α_ radiation (wavelength 0.154 nm) operated at 45 kV and 200 mA in Bragg-Brentano geometry using a K_β_ filter for Cu.

The scanning was in the 2θ range of 10° to 90°, with a step interval of 0.25° and scan speed 10°/min.

As well, the influence of a conductive nanofillers addition on the nanocomposite electrical properties was investigated using a Keithley 2636 source meter.

## 3. Results and Discussion

### 3.1. Influence of BaTiO_3_ Concentration on the Nanogenerator Performance

**Figure 2:**
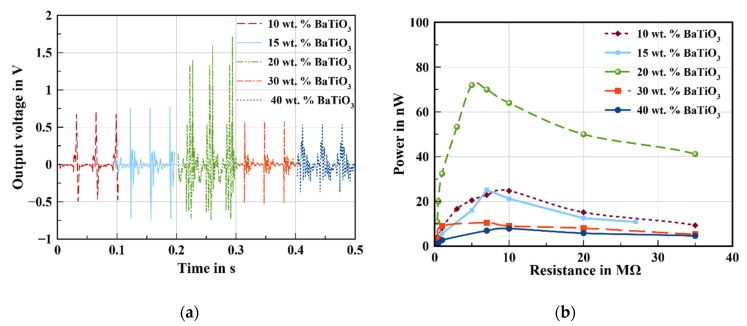
Performance of nanogenerators depending on the concentration of BaTiO_3_: (**a**) output voltage and (**b**) output power.

Upon a repetitive cyclic load, impacting and releasing the NG using a vibration mechanical shaker at 30 Hz, an open output voltage was detected, as illustrated in Figure 2a. The composite material made out of BaTiO_3_/PDMS exhibits typical piezoelectric signals. In fact, when the NG is compressed, a positive voltage pulse is detected where a flow of electrons from the bottom electrode to the upper electrode will be created. Then, when the NG is released, an opposite pulse signal is observed, owing to the recovering of electrons to the original state.

In fact, the piezoelectric voltage output can be expressed by Equation (2):

(2) $$V=\frac{d_{33}}{\varepsilon_{33}}\sigma t$$

where *d*_33_, *ε*_33_, *σ*, and *t* are the piezoelectric coefficient, effective electrical permittivity, mechanical stress change, and film thickness, respectively.

According to Figure 2, the content of BaTiO_3_ greatly affects the output voltage. The nanogenerators containing 20 wt.% BaTiO_3_ exhibit a high output voltage around 1.7 V, as well as a high output power of 72 nW, which is around three times higher than the sample prepared with 10 wt.%.

This can be attributed to the enhanced piezoelectric coefficient of the composite material due to the increased content of piezoelectric particles within the polymer matrix.

Additionally, this enhancement can be also related to the improved young modulus of the composite materials owing to the integration of more ceramic particles.

Kim et al. [26] found that the electric power output of a piezoelectric nanogenerator depends on various physical parameters of the constituent materials, including the piezoelectric coefficient, Young’s modulus, and dielectric constant, where the Young’s modulus plays a crucial role.

In addition, it was noted that, by increasing the amount of BaTiO_3_, the output performance increased first and then reduced after 20 wt.% BaTiO_3_. This reduction is mainly related to the agglomeration of BaTiO_3_ within the polymer matrix and ineffective contact surface area leading to a decreased material mechanical property, as well as piezoelectric property, as demonstrated in Table 2. In fact, the Young’s modulus was increased by doping BaTiO_3_ with PDMS; then, it was reduced at 40 wt.% BaTiO_3_, caused by the inhomogeneous particle’s distribution within the polymer matrix.

**Figure 3:**
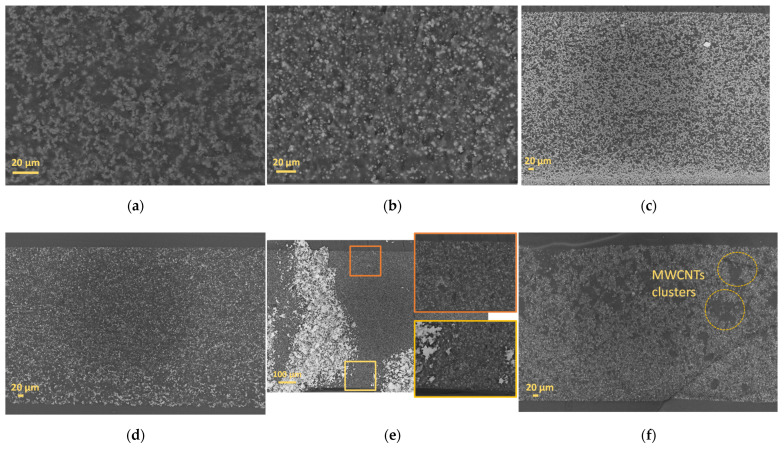

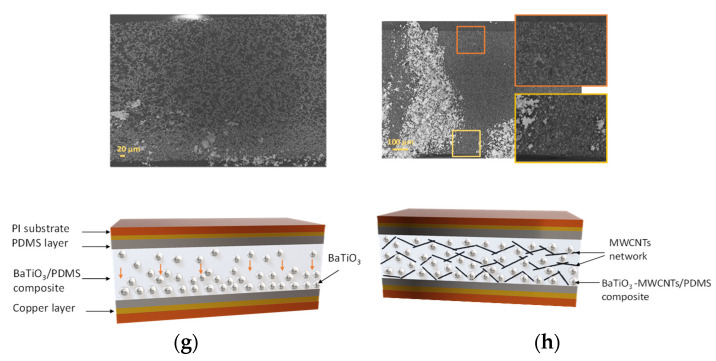
SEM images done in the cross-section for different composites and showing the effects of the addition of MWCNTs: (**a**–**c**) composites with 15 wt.% BaTiO_3_, 20 wt.% BaTiO_3_, and 40 wt.% BaTiO_3_, respectively; (**d**–**f**) 15 wt.% BaTiO_3_/PDMS composite containing 0.3 wt.% MWCNTs, 0.5 wt.% MWCNTs, and 0.75 wt.% MWCNTs, respectively; and (**g**,**h**) illustration of the distribution of BaTiO_3_ in two different cases without and with MWCNTs, respectively.

Figure 3a–c show the cross-sectional morphology images of the 15 wt.% BaTiO_3_/PDMS, 20 wt.% BaTiO_3_/PDMS, and 40 wt.% BaTiO_3_/PDMS, respectively. Those images were taken using SEM to verify the uniformity of particles distribution. According to these figures, BaTiO_3_ particles are not well-distributed within the PDMS matrix and are sedimented at the bottom, leading to the formation of a lot of free space in the upper part, especially for samples prepared with 15 wt.% BaTiO_3_, as demonstrated in Figure 3g. By increasing the amount of BaTiO_3_ within the polymer matrix, less free space between neighboring particles was seen for the sample containing 20 wt.% BaTiO_3_. However, the sedimentation was more pronounced in the 40 wt.% BaTiO_3_ case.

### 3.2. Influence of Addition of MWCNTs on the Nanogenerator Performance

To minimize the free spaces existing in the sample with 15 wt.% BaTiO_3_, the sedimentation of the particles, and to achieve a better performance at a low concentration, MWCNTs were doped within 15 wt.% BaTiO_3_/PDMS with different concentrations.

By the addition of MWCNTs within the composite, enhancement on the BaTiO_3_ particles distribution was remarqued at 0.5 wt.% MWCNTs. In fact, MWCNTs were acting as niches for BaTiO_3_ particles, prompting them to not settle down, as shown in Figure 3d,e,h. In spite of that, increasing the amount of MWCNTs leads to the formation of multiple MWCNTs clusters within the polymer matrix, as shown in Figure 3f.

**Figure 4:**
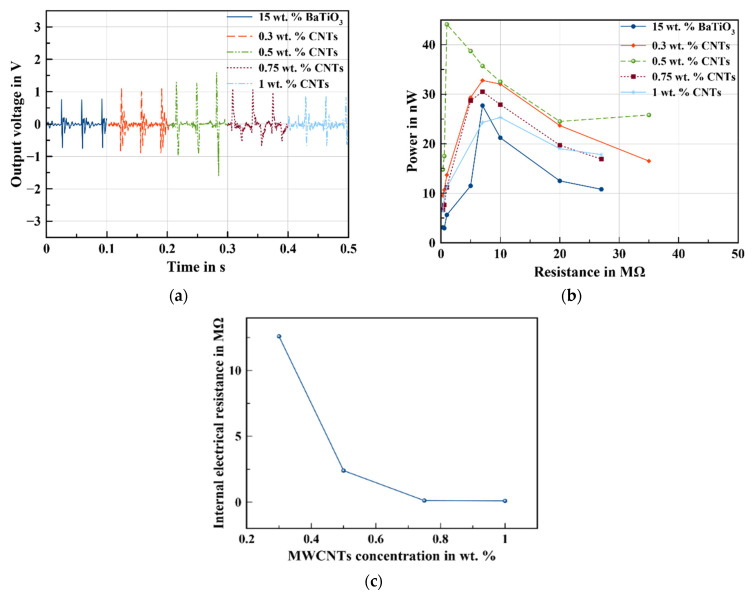
Effect of MWCNT concentration on the performance of the nanogenerators: (**a**) output voltage, (**b**) output power, and (**c**) internal electrical resistance.

The enhancement of piezoceramics particles distribution at low MWCNT concentrations leads to the improvement of the piezoelectric performance of the nanogenerators, as demonstrated in Figure 4a,b.

By evaluating the performance of the nanogenerators containing different amounts of MWCNTs, the important role of MWCNTs was obvious to see. For the composite containing 0.5 wt.% MWCNTs, it was noted that the output voltage was two times higher, as well as the output power. The high output voltage of the NG with MWCNTs compared to the one with only BaTiO_3_ is not only related to the uniform distribution of nanoparticles within the polymer matrix, which is one of the key factors in obtaining a high output voltage, but it is also due to their high electrical conductivity that acts as bridges between piezoceramics nanoparticles and leads at the end to transmit efficiently the electrical charge from the top electrode to the bottom electrode generated during pressing. MWCNTs then act as nanobridges between BaTiO_3_. This effect was suppressed by the presence of only nonconductive PDMS polymers between BaTiO_3_. Similar results were found by Sun et al. [27], the addition of MWCNTs within the ZnO/PDMS composite leads to efficiently enhancing the export of the charge generated by ZnO NPs. Therefore, increasing the amount of CNTs within the composite helps to ensure the self-polarization process within the material owing to the improved electron transport by the formation of conductive pathways within the matrix. According to Figure 4c, the addition of MWCNTs leads to reducing the internal electrical resistance of the nanocomposite where the resistance is sharply minimized at 0.5 wt.% MWCNTs, indicating the formation of the conductive network. By increasing the amount of MWCNTs, the electrical resistance shows a minor reduction, indicating that the 0.5 wt.% presents the percolation threshold of this composite. In fact, many studies show that the dielectric property of composites filled with a conductive filler shows a significant increase near the percolation threshold [28,29]. Additionally, Banerjee et al. [30] illustrated that increasing the MWCNT volume fraction from 1 to 4% within the PZT/epoxy matrix increases the strain coefficient, d_33_, from 0.06 to 0.45 pC/N. This increase can be attributed to the increase in polarization of the composite due to the increased conductivity by the MWCNTs inclusions. In addition, the introduction of MWCNTs can also help to improve the composite mechanical properties, as depicted in Table 2, and ensure a better load transfer within the nanogenerator.

In Reference [31], they demonstrated the synergistic effect between isotropic and anisotropic fillers in constituting a collaborative network that led to enhanced mechanical properties of the composite due to the different aspect ratio and the different surface characteristics, which avoids the filler flocculation phenomenon.

**Figure 5:**
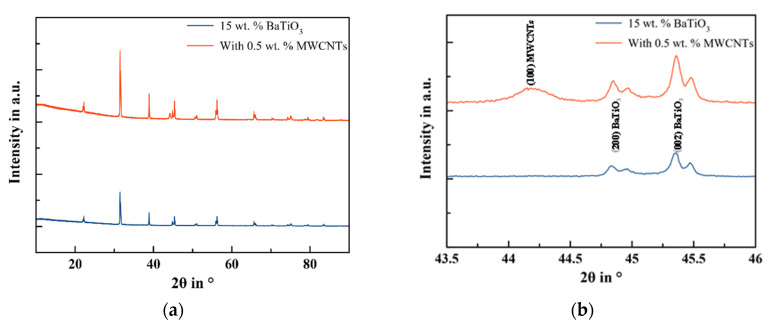
X-ray spectra for different composites with and without MWCNTs: (**a**) spectrum from 2θ = 10° to 90° and (**b**) zoom view in the range of 2θ = 43.5–46°.

It can be seen from the XRD graph presented in Figure 5 that the film prepared with BaTiO_3_ has no other phases except the ones of the BaTiO_3_ phases, indicating that no other impurities are created during processing. In addition, the graph demonstrates sharp peaks, which is a sign of good crystallinity.

The appearance of the peaks (002/200) near 45° and peaks (103/310) near 75° indicate the presence of both crystal phases, which are the tetragonal and cubic phases. As the peaks of PDMS were not interfering with peaks of BaTiO_3_, the Scherrer method for the calculation of particle sizes for single crystallite peaks was adopted as shown in Equation (3) to calculate the particle sizes for samples containing only BaTiO_3_ and the samples with BaTiO_3_ and MWCNTs.

(3) $$D=\frac{K\lambda }{\left(\beta cos\theta \right)}$$

where *λ* is the X-ray wavelength (0.154051 nm), *β* is the full width at half-maximum of (111), and θ is the scattering angle. The measurements show that the particles’ diameters are 1007.36 Å and 955 Å for samples without and with MWCNTs, respectively.

The comparison of the XRD spectrum of composites containing MWCNTs to the spectrum of the sample without MWCNTs shows that the peak intensities of BaTiO_3_ were higher, reflecting the important role of MWCNTs to boost the crystallinity. In fact, the addition of MWCNTs leads to avoid the formation of BaTiO_3_ aggregates and sedimentation that may hinder the nucleation and crystal growth.

### 3.3. Nanogenerator Performance under Simulated Environments

**Figure 6:**
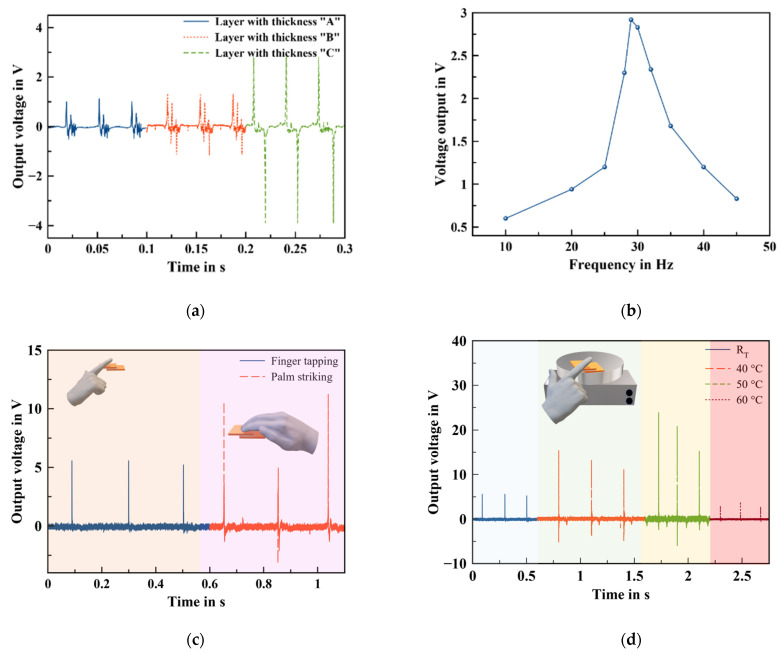
Nanogenerators performances under different conditions: (**a**) output voltage depending on the thickness, (**b**) test under different frequencies from 10 Hz to 45 Hz, (**c**) tapping and striking tests, and (**d**) test under finger tapping and different temperatures.

The performance of the nanogenerator can be tuned as a function of the thickness and applied frequency, as shown in Figure 6a,b. Therefore, three different thicknesses: A, B, and C have been investigated for the nanogenerator under a vibration shaker of 30 Hz, which are 300 µm, 500 µm, and 800 µm, respectively. The study demonstrates that a thicker film shows the highest performance. Additionally, the performance of the optimal nanogenerator geometry was investigated under different force-frequency ranging from 10 Hz to 45 Hz. The study illustrates that, as the external force frequency was rising, the output voltage gradually increased from 0.6 V to 2.93 V at 29 Hz, as depicted in Figure 6b. However, the output voltage was gradually reduced to 0.9 V at 45 Hz. This reduction in the output voltage could be explained by the short time of the compressive force that led to inhibiting the material to recover and returning to its original position before the next force impact.

In order to validate the potential use of the NG for practical applications, the NG was tested under different scenarios, such as finger tapping and palm striking, as illustrated in Figure 6c. The nanogenerator demonstrates the ability to harvest biomechanical energy efficiently up to 5.56 V and 11.22 V under finger and palm striking, respectively.

One of the important things that needs to be addressed is the effect of the environmental changes on the performances of the nanogenerator. Herein, the effect of a temperature change on the NG performance was examined from room temperature up to 60 °C. The measurements were conducted by placing the nanogenerator on top of the hotplate for 10 min. Then, a compressive force was applied by finger tapping. Figure 6d shows the output voltages at different temperatures. It is noted that the output voltage increased until 50 °C from 5.56 V to 23.86 V. Then, the NG exhibited a decrease in the output voltage to reach 3.74 V. The increase can be explained by the enhanced electrical properties of the nanocomposite. In fact, MWCNTs exhibit a negative temperature coefficient (NTC) behavior, due to their semi-conductive behavior. By increasing the temperature, the electrons’ mobility will be improved, which facilitates the electron tunneling effect, resulting in an increase in conductivity. Additionally, the mobility of polymer molecular chains will be increased, leading to expansion of the nanocomposite, which can boost the distribution of piezoceramics within the polymer matrix. In this way, the nanogenerator electromechanical coupling coefficient will be increased with the temperature. However, the decreasing at 60 °C may be due to the destruction of the conductive network caused by the thermal expansion of the polymer, which inhibited the electron transfer. In fact, the expansion of the polymer will increase the tunneling resistance between neighboring MWCNTs, leading to a sharp reduction of the conductivity, as well to creation of free spaces between ceramic particles.

## 4. Conclusions

In this work, eco-friendly and cost-effective flexible nanogenerators were developed using a solution mixing method followed by mold casting. The prepared nanogenerators are based on lead-free piezoelectric BaTiO_3_ nanoparticles known by their high piezoelectric coefficient. The results show that the performance of nanogenerators containing only BaTiO_3_ particles depends greatly on the concentration of BaTiO_3_. In fact, increasing the BaTiO_3_ concentration improves the output voltage and power, owing to the enhanced piezoelectric coefficient of the composite material. However, excessive BaTiO_3_ leads to the reduction of the composite performance, caused by the lack of homogeneity and the sedimentation of the particles at the bottom.

To boost the performance of the BaTiO_3_/PDMS composite, MWCNTs as conductive elements were incorporated within the composite.

Thereby, the BaTiO_3_ particles were better and homogeneously dispersed in the PDMS matrix, and the sedimentation of the particles was avoided. Additionally, the addition of MWCNTs contributes to the improvement of the electrical and mechanical properties of the composite, especially at the percolation threshold. This later significantly enhanced the performance of the NG, which became two times higher compared to the NG without MWCNTs.

The study has also shown the importance of geometrical parameters such as the composite thickness and the test conditions on the output performance.

Increasing the composite thickness leads to enhancing the output voltage, as well as the applied frequency.

The optimized NG structure favors the scavenging of biomechanical energy without any additional poling process around 5.56 V under gentle finger tapping and 11.22 V under palm striking. Therefore, the realized NG offers great opportunities for achieving wearable energy harvesters for self-powered electronics.

This work demonstrates the importance of addressing the temperature aspect on the NG response. The results showed that the output voltage increased enormously by increasing the temperature. As a consequence, several aspects should be deeply investigated in the future in regard to the environmental effects.

## Figures and Tables

**Table 1:** Summary of the composites compositions and dimensions realized in this work.

Type of Composite	BaTiO_3_ Concentration	MWCNTs Concentration	Composite Thickness
Without MWCNTs	10 wt.%	-	500 μm
	15 wt.%	-	
	20 wt.%	-	
	30 wt.%	-	
	40 wt.%	-	
With MWCNTs	15 wt.%	0.3 wt.%	500 μm
		0.5 wt.%	300, 500, 800 μm
		0.75 wt.%	500 μm
		1 wt.%	500 μm

**Table 2:** Young’s modulus for different composites.

Composite Composition	Young’s Modulus (MPa)
15 wt.%	0.08
20 wt.%	0.18
30 wt.%	0.24
40 wt.%	0.19
15 wt.% + 0.3 wt.% MWCNTs	0.09
15 wt.% + 0.75 wt.% MWCNTs	0.23

## Data Availability

Not applicable.
